# Membrane-Anchored Mobile Tethers Modulate Condensate Wetting, Localization, and Migration

**DOI:** 10.1103/kxpb-9srd

**Published:** 2026-05-15

**Authors:** Qiwei Yu, Trevor GrandPre, Andrew G. T. Pyo, Andrej Košmrlj, Ned S. Wingreen

**Affiliations:** 1 Lewis-Sigler Institute for Integrative Genomics, Princeton University, Princeton, New Jersey 08544, USA; 2 Department of Physics, Princeton University, Princeton, New Jersey 08544, USA; 3 Department of Physics and Center for Biomolecular Condensates, Washington University in St. Louis, St. Louis, Missouri 63130, USA; 4 Department of Applied Physics, Stanford University, Stanford, California 94305, USA; 5 Department of Mechanical and Aerospace Engineering, Princeton University, Princeton, New Jersey 08544, USA; 6 Princeton Materials Institute, Princeton University, Princeton, New Jersey 08544, USA; 7 Department of Molecular Biology, Princeton University, Princeton, New Jersey 08544, USA

## Abstract

Biomolecular condensates frequently rely on membrane interactions for recruitment, localization, and biochemical substrates. Many of these interactions are mediated by membrane-anchored molecules such as proteins or specific lipids, which we refer to as “mobile tethers” since they can typically diffuse within the membrane while still interacting with the condensate. The presence of mobile tethers creates a surface with dynamic and spatially inhomogeneous wetting properties that are typically overlooked by traditional wetting theories. Here, we propose a general theoretical framework to study how mobile tethers impact both equilibrium and dynamic properties of condensate wetting. We show that a favorable tether-condensate interaction leads to tether enrichment at the condensate-membrane interface, which modifies the equilibrium condensate–membrane surface tension and contact angle. Increasing tether abundance on the membrane can drive transitions between wetting regimes, with only a modest tether density and binding energy required for biologically relevant scenarios. Furthermore, tethers modulate how condensates react to complex membrane geometries. By helping condensates coat membranes, mobile tethers can facilitate condensate localization to junctions of membrane structures, such as the reticulated membranes inside the algal pyrenoid. Both tether abundance and mobility affect how droplets interact with complex membrane geometries, such as droplet migration along membrane tubules of varying radii. These results provide a framework to study the implications of tether-mediated condensate-membrane interactions for cellular organization and function.

## I. INTRODUCTION

Biomolecular condensates—intracellular compartments formed via phase separation—are essential for diverse biological processes, including gene regulation, metabolism, and cell signaling [1,2]. In many instances, proper condensate function relies on interactions with membranes [3–9]. These membrane interactions can spatially organize condensates, concentrate interaction partners, and facilitate access to reactants. The algal pyrenoid exemplifies this interplay [10]: condensates enriched with the CO_2_-fixing enzyme Rubisco form around traversing membranes that supply CO_2_ to enhance photosynthetic efficiency. Conversely, condensates can also facilitate membrane processes such as transport, signaling, force generation, and structural remodeling. For example, focal adhesion kinase forms condensates on the cytoplasmic membrane, binding to lipids at sites where focal adhesions assemble, thereby regulating cell motility [11]. Similarly, B-cell activation involves condensation on the plasma membrane that is essential for downstream signaling [12]. More broadly, unraveling the dynamic relationship between condensates and membranes is proving to be essential for understanding intracellular organization and function.

In many cases, membrane-associated condensates do not directly wet membranes. Instead, they adhere to membrane surfaces via tethering molecules, such as proteins or specific lipids, that are anchored to the membrane. In the pyrenoid of the model alga *Chlamydomonas reinhardtii*, for example, pyrenoid-traversing membranes feature tethers like RBMP1, RBMP2, and SAGA1, which directly bind to Rubisco [13,14]. These tether proteins are essential for the assembly of the pyrenoid condensate around traversing membrane tubules, a structure that is crucial for the pyrenoid’s function in CO_2_ fixation. In this case and others, elucidating how tethers mediate condensate-membrane interactions is key to understanding the structure and function of membrane-associated condensates.

A key characteristic of these tether molecules is their ability to diffuse laterally within the membrane. As the condensate wets the membrane, the tethers can dynamically redistribute, enriching at the condensate-membrane interface due to favorable interactions with the condensate. This creates a surface with dynamic and spatially inhomogeneous wetting properties, which can affect both the equilibrium and the dynamic aspects of condensate wetting. These effects are typically overlooked by traditional wetting theories, which often assume static surface properties [15,16], or theories of soft wetting, where the dynamics comes from substrate deformation [17]. Here, motivated by both biological significance and theoretical interest, we seek to address the general question of how mobile tethers affect the condensate-membrane interaction and wetting.

In this work, we present a general theoretical framework that describes the coupled dynamics of condensates and mobile tethers. We find that mobile tethers enrich in the condensate-membrane interface, thereby reducing the surface tension with the membrane and modifying the equilibrium contact angle. By tuning the expression level of attractive tethers, cells can drive transitions from nonwetting to partial or complete wetting. The per-tether binding energy required for such wetting transitions is estimated to be modest (only a few $k_{\text{B}}T$) for typical values of tether density and condensate surface tension. Furthermore, mobile tethers facilitate condensate localization to intersecting membrane structures, such as the reticulated membranes inside the pyrenoid. Finally, both tether abundance and mobility affect droplet migration on spatially varying membrane structures such as tapering tubules. Overall, our framework provides tools for understanding the role of tether-mediated condensate-membrane interactions in cellular organization and function.

## II. RESULTS

### A general theoretical framework for tether-mediated wetting

A.

We study a general theory that describes the densities of tethers and condensates with fields ψ (defined on the membrane) and ϕ (defined in the bulk), respectively. A high (low) value of ϕ corresponds to a condensate dense (dilute) phase. The interactions are captured by a total free energy:

(1) $$\beta F=c_{\psi ,0}\int \text{d}A\left[f_{\psi }(\psi )+\frac{\lambda_{\psi }}{2}(\nabla \psi {)}^{2}-E\left(\psi ,{\left.\phi \right|}_{\text{surf}}\right)\right]\;+c_{\phi ,0}\int \text{d}V\left[f_{\phi }(\phi )+\frac{\lambda_{\phi }}{2}(\nabla \phi {)}^{2}\right],$$

where the first integral is over the membrane area, and the second integral is over the bulk volume. Energy is measured in units of $\beta^{-1}=k_{\text{B}}T$. $c_{\psi ,0}$ and $c_{\phi ,0}$ are reference concentrations for the tether and condensate so that the free-energy densities are nondimensionalized: $E\left(\psi ,{\left.\phi \right|}_{\text{surf}}\right)$ captures both condensate-tether and condensate-membrane interactions; $f_{\psi }(\psi )$ and $f_{\phi }(\phi )$ are the free-energy densities of tethers and condensates, respectively; and $\lambda_{\psi }$ and $\lambda_{\phi }$ are constants associated with interface energies. Without loss of generality, we consider the nondimensionalized system, setting $c_{\psi ,0}k_{\text{B}}T=1$ and $c_{\phi ,0}k_{\text{B}}T=1$ by rescaling the unit of length by $l_{0}=c_{\psi ,0}/c_{\phi ,0}$ and energy by $E_{0}=c_{\phi ,0}k_{\text{B}}Tl_{0}^{3}$ (see Sec. I A of the Supplemental Material [18] for details). The model encompasses a large class of systems and interactions by allowing the free-energy densities $f_{\psi }(\psi )$ and $f_{\phi }(\phi )$ and the interaction energy $E\left(\psi ,{\left.\phi \right|}_{\text{surf}}\right)$ to take any reasonable form.

**FIG. 1.**
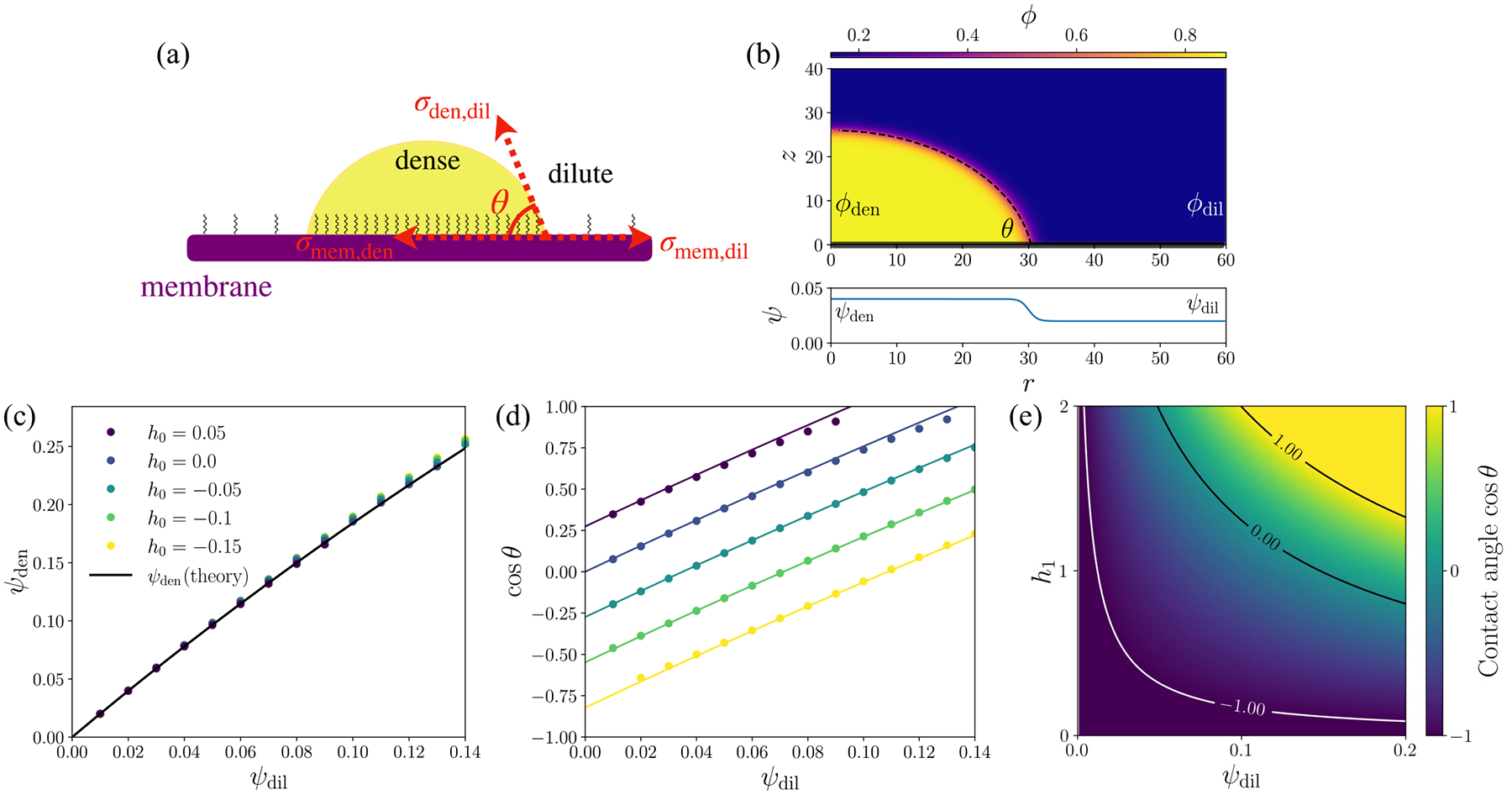
Mobile-tether-mediated condensate wetting of membranes. (a) Illustration of a biomolecular condensate (yellow) interacting with mobile tether molecules (black) to wet a membrane (purple). The interaction creates a localized enrichment of tethers around the condensate, surrounded by a lower background concentration of tethers. mem, den, and dil represent membrane, dense phase, and dilute phase, respectively. The contact angle θ is determined by force balance at the three-phase junction: $\sigma_{\text{den},\text{dil}}\;\text{cos}\;\theta =\sigma_{\text{mem},\text{dil}}-\sigma_{\text{mem},\text{den}}$, where the σ’s are surface tensions. (b) A typical equilibrium concentration profile obtained from numerical simulations. The condensate field ϕ (top) and the tether field ψ (bottom) are plotted in cylindrical coordinates (r,z) with axial symmetry. The thick black line indicates the flat membrane at z=0. The black dashed curve is a spherical cap fit to the condensate surface contour. (c) Condensate-enriched tether concentration $\psi_{\text{den}}$ increases with bulk tether concentration $\psi_{\text{dil}}$, for different condensate-membrane interactions $h_{0}$, consistent with theory [solid curve, Eq. (4)]. (d) Contact angle cosθ as a function of tether concentration $\psi_{\text{dil}}$ for different values of $h_{0}$ [see legend in panel (c)] agrees well with theory [solid curves, Eq. (5)]. (e) cosθ [Eq. (5)] as a function of condensate-tether interaction $h_{1}$ and tether concentration $\psi_{\text{dil}}$ for $h_{0}=-0.2$. Contours for cosθ=±1 represent wetting transitions to complete and no wetting, respectively. In all simulations, ψ has a Dirichlet boundary condition while ϕ has a no-flux boundary condition. Tethers are purely entropic $\left(\chi_{\psi }=0\right)$; see Fig. S1 [18] for simulations with $\chi_{\psi }\neq 0$. For panels (b)–(d), tether-condensate interaction strength is $h_{1}=1$; see Fig. S2 [18] for other values of $h_{1}$. Other parameters are $\chi_{\phi }=2.5,\lambda_{\phi }=1,\lambda_{\psi }=0;\;\psi_{\text{dil}}=0.02$ and $h_{0}=0$ for panel (b); and $h_{0}=-0.2$ for panel (e).

By minimizing the free energy in Eq. (1), we obtain the equilibrium concentration profile, from which the contact angle θ can be measured [Figs. 1(a) and 1(b)]. To study the dynamics of wetting, we can further prescribe conserved (model B) dynamics [23]:

(2) $$\partial_{t}\psi =\nabla \cdot \left(M_{\psi }\nabla \mu_{\psi }\right),\;\partial_{t}\phi =\nabla \cdot \left(M_{\phi }\nabla \mu_{\phi }\right),$$

where $M_{\psi }$ and $M_{\phi }$ are mobility coefficients, and $\mu_{\psi }=\delta F/\delta \psi$ and $\mu_{\phi }=\delta F/\delta \phi$ are the dimensionless chemical potentials of the tethers (ψ) and the condensate (ϕ), respectively. We rescale time by $t_{0}=l_{0}^{2}/\left(M_{\phi }E_{0}\right)$ such that $M_{\phi }=1$. The model B approach describes overdamped diffusive relaxation of the condensate and tether fields and does not include hydrodynamic momentum transport, which would require model H [23].

To illustrate the physical picture, we study a minimal model for interrogating how mobile tethers affect condensate wetting. We consider a linear interaction energy, $E(\psi ,\phi )=\left(h_{0}+h_{1}\psi \right)\phi$, where $h_{0}$ and $h_{1}$ describe condensate-membrane and condensate-tether interactions, respectively. $h_{1}>0$ represents an attractive condensate-tether interaction, while the bare membrane could be either repelling $\left(h_{0}<0\right)$ or attracting $\left(h_{0}>0\right)$ for the condensate. We use Flory-Huggins free energies for self-energies $f_{\xi }\left(\xi \right)=\xi \;\text{ln}\;\xi +(1-\xi )\text{ln}(1-\xi )+\chi_{\xi }\xi (1-\xi )$, with ξ∈{ψ,ϕ} representing the area or volume fraction of tether or condensate, respectively [24]. The first two terms represent the entropy of mixing, while the parameter $\chi_{\xi }$ quantifies the effective interaction bias between components: positive $\chi_{\xi }$ corresponds to unfavorable mixing and promotes phase separation. In the limit $\chi_{\psi }=0$ (purely entropic limit for tethers), the self-organization of tethers in the membrane is energetically neutral and driven purely by entropy.

Regarding how tether mobility affects condensate behavior, we find that the physical picture that emerges from the minimal model persists over a broad range of physically motivated parameter choices and modeling details, such as condensate free-energy density, tether-tether interaction, or tether-condensate interaction strength (see Sec. I C of the Supplemental Material [18]). It is also straightforward to extend the model to describe multicomponent condensates and/or tethers, as well as more complex interactions.

### B. Mobile tethers control equilibrium wetting properties

In classical wetting theory, the contact angle θ of a droplet on a surface is determined by force balance at the three-phase junction through the Young-Dupré equation [15], which relates θ to the difference of surface tensions [Fig. 1(a)]. In the presence of mobile tethers, favorable tether-condensate interactions enrich tethers within a wetting condensate [Fig. 1(a)], thereby creating a surface with inhomogeneous wetting properties, which in turn modifies the surface tensions and the contact angle.

Condensate phase separation creates dense and dilute phases in the bulk, with binodal concentrations $\phi_{\text{den}}$ and $\phi_{\text{dil}}$ (as measured in volume fractions), respectively. The concentration difference $\Delta \phi =\phi_{\text{den}}-\phi_{\text{dil}}$ drives the attraction of tethers to the condensate, resulting in a tether area fraction, $\psi_{\text{den}}$, in contact with the dense phase, which is higher than that in contact with the dilute phase $\psi_{\text{dil}}$ [Fig. 1(b)]. This partition of tethers reaches equilibrium when chemical potentials are balanced: $\mu_{\psi }\left(\psi_{\text{den}},\phi_{\text{den}}\right)=\mu_{\psi }\left(\psi_{\text{dil}},\phi_{\text{dil}}\right)$, which leads to

(3) $$\text{ln}\frac{\psi_{\text{den}}}{1-\psi_{\text{den}}}=\text{ln}\frac{\psi_{\text{dil}}}{1-\psi_{\text{dil}}}+h_{1}\Delta \phi +2\chi_{\psi }\left(\psi_{\text{den}}-\psi_{\text{dil}}\right),$$

where we have approximated the condensate concentrations at the surface with the bulk binodal concentrations (see Sec. I B of the Supplemental Material [18] for details). Equation (3) is accurate to first order in the interaction energy $O\left(h_{0},h_{1}\right)$. If the tethers mix in the membrane purely entropically $(\chi_{\psi }=0),\;\psi_{\text{den}}$ can be solved analytically:

(4) $$\psi_{\text{den}}=\frac{\psi_{\text{dil}}e^{h_{1}\Delta \phi }}{1+\psi_{\text{dil}}\left(e^{h_{1}\Delta \phi }-1\right)},$$

which can be viewed as Langmuir adsorption of tethers by the condensate [25]. Equation (4) agrees well with numerical simulations across a wide range of $\psi_{\text{dil}}$, for both repelling $\left(h_{0}<0\right)$ and attracting $\left(h_{0}>0\right)$ interactions between the bare membrane and the condensate [Fig. 1(c)].

The presence of attractive tethers reduces both surface tensions $\sigma_{\text{mem,den}}$ and $\sigma_{\text{mem,dil}}$. However, the decrease in $\sigma_{\text{mem,den}}$ is more substantial due to tether enrichment in the condensate $\left(\psi_{\text{den}}>\psi_{\text{dil}}\right)$. This, in turn, modifies the contact angle θ, which is determined by force balance at the three-phase junction: $\sigma_{\text{den},\text{dil}}\;\text{cos}\;\theta =\sigma_{\text{mem},\text{dil}}-\sigma_{\text{mem},\text{den}}$. To the leading order in $O\left(h_{0},h_{1}\right)$, the modified contact angle is (see Sec. I B of the Supplemental Material [18] for details)

(5) $$\text{cos}\;\theta =\frac{\sigma_{\text{mem},\text{dil}}-\sigma_{\text{mem},\text{den}}}{\sigma_{\text{den},\text{dil}}}=\frac{\Delta \sigma_{0}+\Delta \sigma_{1}}{\sigma_{\text{den},\text{dil}}},$$

where $\Delta \sigma_{0}=h_{0}\Delta \phi$ is the surface tension difference in the absence of tethers, and $\Delta \sigma_{1}=\text{ln}\frac{1-\psi_{\text{dil}}}{1-\psi_{\text{den}}}-\chi_{\psi }\left(\psi_{\text{den}}^{2}-\psi_{\text{dil}}^{2}\right)$ is the additional surface tension difference due to mobile tethers. In the purely entropic limit for tethers ($\chi_{\psi }=0),\;\Delta \sigma_{1}$ simplifies to

(6) $$\Delta \sigma_{1}=\text{ln}\left[1+\psi_{\text{dil}}\left(e^{h_{1}\Delta \phi }-1\right)\right],$$

which increases monotonically with tether abundance $\psi_{\text{dil}}$ and tether-condensate interaction $h_{1}$. Indeed, numerical simulations find the contact angle in simulations to be in excellent agreement with Eq. (5) [Fig. 1(d), solid curves]. Thus, an attractive interaction due to mobile tethers can substantially modulate wetting over a wide range of contact angles.

Wetting transitions occur at cosθ=1, when a droplet completely wets a membrane, and at cosθ=-1, when a droplet detaches from a membrane (nonwetting). Tethers can induce transitions between these wetting regimes. This can be simply illustrated in the limit of purely entropic tethers $\left(\chi_{\psi }=0\right)$: For a repelling membrane that is initially in the nonwetting regime $\left(h_{0}<-\sigma_{\text{den},\text{dil}}/\Delta \phi \right)$, both partial wetting [cosθ∈(-1,1)] and complete wetting (cosθ=1) regimes can be achieved via a high enough density of attractive tethers [Fig. 1(e)]. To reach complete wetting, the required critical density of tethers is $\psi_{\text{dil}}^{⋆}=\frac{e^{\sigma_{\text{den},\text{dil}}-h_{0}\Delta \phi }-1}{e^{h_{1}\Delta \phi }-1}$, which must stay below 1 since ψ is defined in terms of area fraction. Since $\psi_{\text{dil}}^{⋆}$ vanishes in the limit of large $h_{1}$, a finite density of tethers is sufficient to access all three wetting regimes as long as the tether-condensate attraction is strong enough.

For the sake of analytical tractability, our comparison of theoretical expressions to numerical simulations (Fig. 1) has primarily focused on purely entropic mobile tethers ($\chi_{\psi }=0$). However, our theory can also describe tethers with a nonzero energetic cost of mixing $\left(\chi_{\psi }\neq 0\right)$ and incorporate alternative forms of condensate free energies $f_{\phi }(\phi )$. These generalizations conform with the same physical picture, with excellent quantitative agreement between theory and simulations (see Sec. I C of the Supplemental Material [18]).

For real tether molecules, how much binding energy is required to significantly affect wetting properties? Typically, the membrane would be slightly repulsive for polymer condensates because being close to a membrane reduces the conformational entropy of polymers, leading to an estimated $\Delta \sigma_{0}\approx -10^{-1}k_{\text{B}}T/{\text{nm}}^{2}$ [26]. In aqueous buffer, biomolecular condensate surface tensions are typically of the same order, $\sigma_{\text{den,dil}}\approx 10^{-1}k_{\text{B}}T/{\text{nm}}^{2}$ [27]. Thus, to drive wetting, tethers must reduce surface tension by the same order, $\Delta \sigma_{1}\approx 10^{-1}k_{\text{B}}T/{\text{nm}}^{2}$. A typical tether density of $n\approx 10^{-2}\;{\text{nm}}^{-2}$ [28] yields a required binding energy of $\epsilon \approx O(1)k_{\text{B}}T$ (see Sec. II of the Supplemental Material [18] for details). Despite being a rough estimate, these calculations show that a modest per-tether binding energy (a few $k_{\text{B}}T$) is sufficient to drive wetting transitions. Therefore, cells can potentially regulate condensate wetting by tuning the expression level of tether molecules.

### C. Mobile tethers facilitate condensate localization dynamics

Thus far, we have focused on equilibrium morphologies. How might mobile tethers affect the dynamics of condensate formation and localization? In the alga *C. reinhardtii*, for example, the pyrenoid condensate dissolves and reforms every cell division [29], and the new pyrenoid centers around a reticulated region where many membrane tubules meet. Since the reticulated region has a high membrane area per volume, it might therefore be able to enrich tethers more effectively than other regions of the tubule. Hence, we hypothesize that mobile tethers may facilitate condensate localization by enrichment in the reticulated region.

**FIG. 2.**
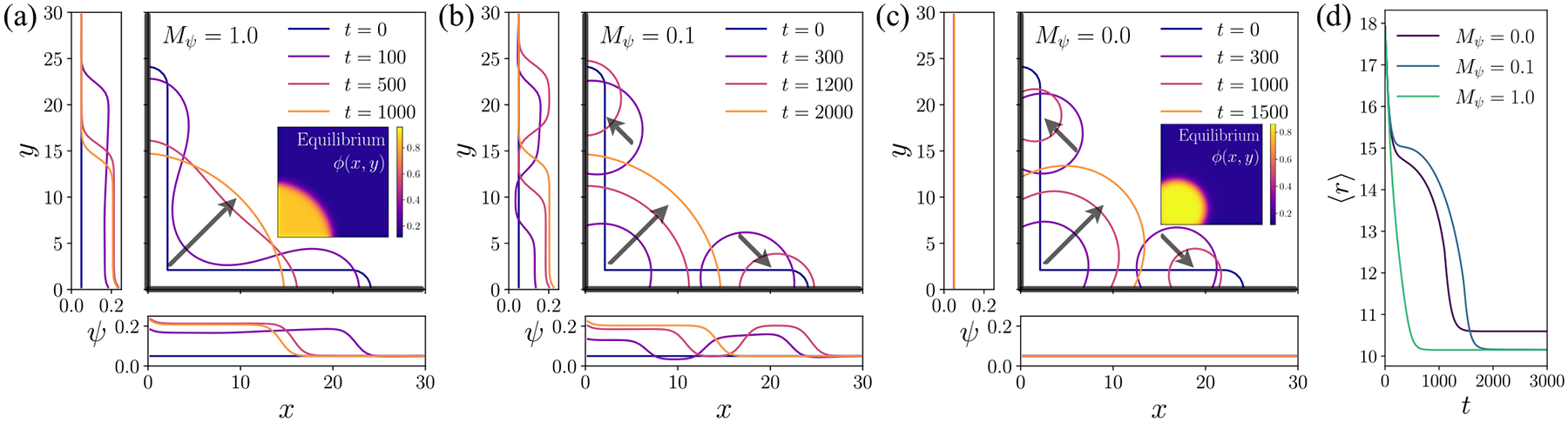
Mobile tethers facilitate dynamic condensate relocalization. (a)–(c) Dynamics of condensate localization for tether mobility $M_{\psi }=1.0$ (a), $M_{\psi }=0.1$ (b), and $M_{\psi }=0.0$ (c). The simulation domain is a two-dimensional system (x,y) with membranes on the left and bottom boundaries (indicated by thick black lines). Different colors indicate concentration profiles at different times (legend), with the condensate ϕ represented by interface contours and the tether density ψ shown in the left and bottom insets. Insets in panels (a) and (c) show the final equilibrium profile for ϕ(x,y). Black arrows indicate the time evolution of the interface contours to guide the eye. The tether density at the boundaries is $\psi_{\text{dil}}=0.05$. The overall ⟨ϕ⟩ is conserved due to no-flux boundary conditions. (d) Condensate location as quantified by the average distance from the bottom-left corner $\langle r\rangle =\int \delta \phi (x,y)\sqrt{x^{2}+y^{2}}\text{d}x\text{d}y/\int \delta \phi (x,y)\text{d}x\text{d}y$, where $\delta \phi =\phi -\phi_{\text{dil}}$. See the Supplemental Material [18] for details and simulation videos.

To simply illustrate this mechanism, we study a two-dimensional system which is bounded by membranes on the left and bottom sides and closed (by no-flux boundary conditions) on the other two (Fig. 2). The bottom-left corner is most favorable for the condensate since it can interact there with the largest amount of membrane area (and therefore tethers), analogous to the reticulated region in the pyrenoid. We envision that the condensate can form near the membrane via either heterogeneous nucleation or recruitment by membrane proteins. Thus, initially in simulations, the condensate coats part of the membrane, and its bulk concentration is between binodal and spinodal concentrations. We then simulate the model to study how tether mobility affects condensate localization dynamics.

If tethers have a high mobility $\left(M_{\psi }=1\right)$, they quickly enrich in the condensate and help it localize to the corner [Fig. 2(a)]. In contrast, if the tether mobility is low $\left(M_{\psi }=0.1\right)$, the condensate first breaks up into smaller droplets and only slowly relocalizes to the corner through a coarsening process [Fig. 2(b)]. In both cases, the coarsening is purely due to diffusive flux of the condensate (Ostwald ripening). Even though both cases reach the same equilibrium state, the latter process is much slower [Fig. 2(d)]. If the tethers are completely immobile ($M_{\psi }=0$, which is equivalent to a homogeneous membrane without tethers), the condensate still localizes to the membrane junction, although the equilibrium state is slightly different due to a different contact angle [Fig. 2(c), inset]. The localization dynamics are comparable to those of the low-tether-mobility system and much slower than those of the high-tether-mobility one [Figs. 2(c) and 2(d)]. Thus, by helping the condensate to optimize its membrane contacts, mobile tethers can facilitate coarsening and localization with respect to membrane structures.

### D. Tether abundance and mobility affect condensate migration on tubules

Our theoretical framework enables the study of mobile-tether-mediated wetting of a myriad of possible membrane structures, including tubes, sheets, and cristae. Membrane morphology can also vary in space: For example, membrane structures in the pyrenoid condensate in *C. reinhardtii* transition from flat sheets to cylindrical tubules to even narrower contorted tubules as they traverse the condensate [13]. As droplets wet such spatially varying structures, they may be impelled to migrate along the surface to minimize the overall energy [30]. As highlighted in the example above, the presence of mobile tethers could modulate or amplify the effects of membrane geometry on condensate behavior, such as condensate migration.

**FIG. 3.**
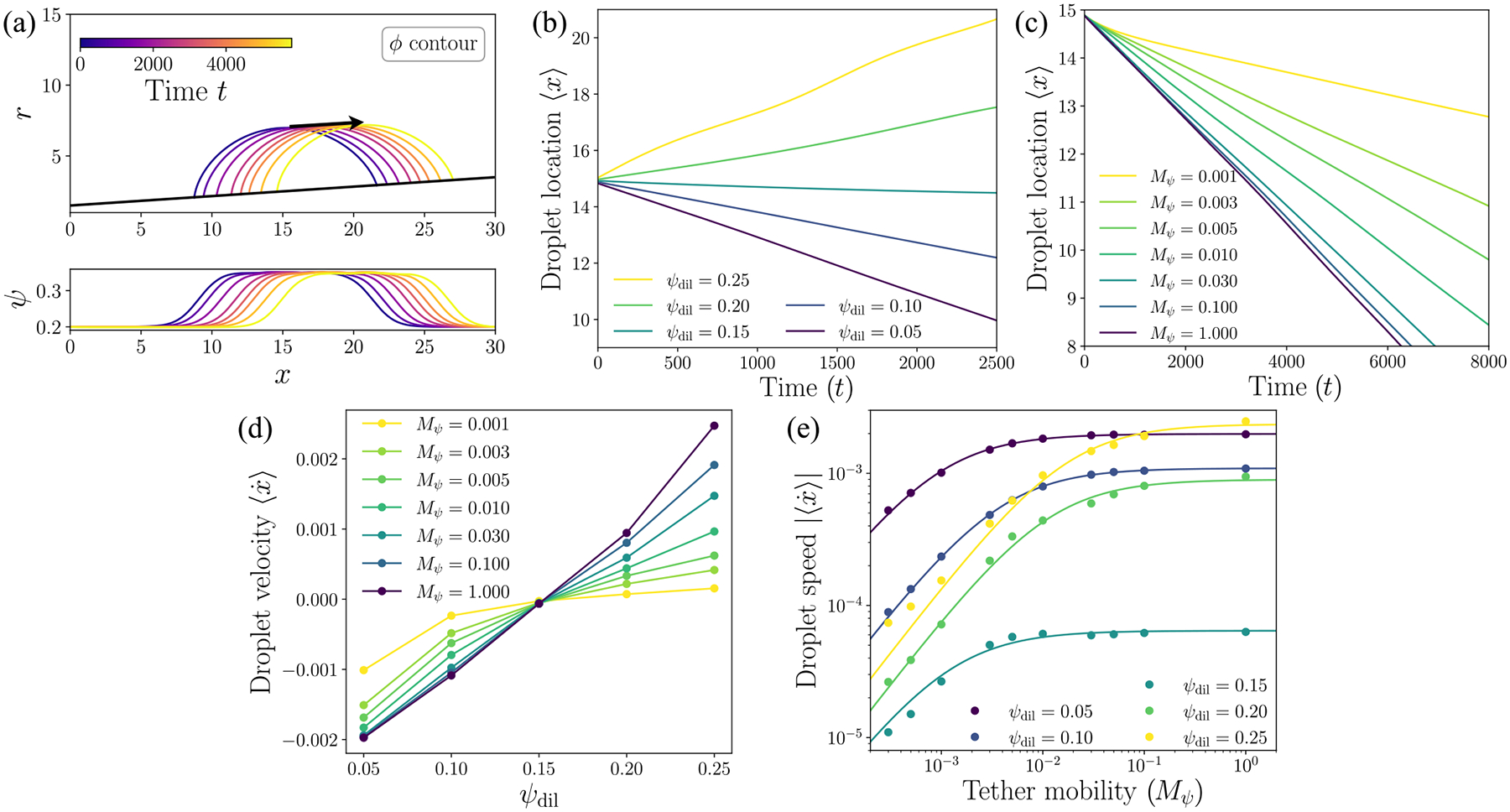
Mobile tethers affect condensate migration on a tubule of varying radius. Here, we consider a (truncated) cone geometry where the tubule radius varies linearly along its long axis, although similar results hold for other geometries as well. (a) Time course of the condensate ϕ (top contours) and tether ψ (bottom) densities on a tubule of varying radius, in cylindrical coordinates (r,x) where x runs along the central axis of the tubule. Curves of different colors represent different times (inset legend), with the arrow indicating the direction of migration. The black line indicates the tubule surface. (b) and (c) Condensate location as quantified by the average position ⟨x⟩ for different tether concentrations $\psi_{\text{dil}}$ for $M_{\psi }=1.0$ (b) and different tether mobilities $M_{\psi }$ for $\psi_{\text{dil}}=0.10$ (c). (d) Migration velocity $\langle \dot{x}\rangle \equiv \frac{\text{d}\langle x\rangle }{\text{d}t}$ as a function of $\psi_{\text{dil}}$ for different values of $M_{\psi }$. (e) Migration speed $\left|\langle \dot{x}\rangle \right|$ as a function of $M_{\psi }$ for different concentrations of $\psi_{\text{dil}}$. Solid curves are fits to $|\langle \dot{x}\rangle |={\left(f_{\phi }+f_{\psi }/M_{\psi }\right)}^{-1}$, with $f_{\phi }$ and $f_{\psi }$ being fitting parameters. See the Supplemental Material [18] for details and parameters.

To illustrate such geometric effects, we consider the dynamics of a condensate that wets a membrane tubule of varying radius. Such structures can be found in the pyrenoid, where the traversing tubules narrow as they go inside the condensate [31]. Here we consider a (truncated) cone geometry where the tubule radius varies linearly along its long axis [Fig. 3(a), black line], although the theoretical arguments are general for other geometries as well. When the tubule is thin (compared to $V^{1/3}$, where V is the droplet volume), the droplet can adopt an axisymmetric barrel-like shape that wraps around the tubule. By contrast, the droplet can also wet only one side of the tubule and adopt an asymmetric clamshell-like shape when the tubule is thick [32–35]. Here, we focus on the former case, where the droplet is able to wrap around the tubule [Fig. 3(a)].

We expect such an axisymmetric droplet to migrate along the tubule, moving down the gradient of free energy until reaching a minimum-energy equilibrium position. The equilibrium location will depend on the contact angle θ, where a smaller θ (more wetting) favors regions of larger radius, and vice versa. By approximating the cross section of the barrel-shaped droplet as circular (see Sec. III of the Supplemental Material [18] for numerical justification), we find that the droplet always moves to the smallest radius for θ>π/2, while for θ<π/2 the droplet prefers a finite radius that scales as $r\sim V^{1/3}\text{cot}\;\theta$ (see Sec. III of the Supplemental Material [18] for details). We note, however, that if $r/V^{1/3}$ is too large, the axisymmetric barrel becomes unstable and the droplet moves to wet only one side of the cylinder (clamshell shape) [32,35]. Nevertheless, for a droplet initialized on a relatively thin tubule, the contact angle θ dictates whether it initially moves to a small radius or a large radius.

Since the contact angle θ can be modulated by the tether abundance $\psi_{\text{dil}}$ [Eq. (5), Fig. 1(d)], we expect that $\psi_{\text{dil}}$ can affect the equilibrium location of the droplet on the tubule. Specifically, increasing the tether abundance $\psi_{\text{dil}}$ decreases θ [Fig. 1(d)], thereby shifting the equilibrium location to a larger radius. Indeed, when we initialize a droplet at a particular location on the tubule, it migrates towards a small radius when $\psi_{\text{dil}}$ is low (large θ), but towards a large radius when $\psi_{\text{dil}}$ is high (small θ) [Fig. 3(b)]. Increasing tether mobility $M_{\psi }$ leads to faster migration [Fig. 3(c)], while a very small $M_{\psi }$ can lead to self-trapping, pinning the droplet and arresting migration.

These results suggest that tether abundance and mobility affect different aspects of droplet migration on spatially varying membrane structures: Tuning the tether abundance $\psi_{\text{dil}}$ can modulate the total force on the droplet and control its preferred localization on the tubule, while tuning the tether mobility $M_{\psi }$ can control droplet migration speed [Fig. 3(d)]. In the overdamped limit, the driving force due to the free-energy gradient (or equivalently, surface tension forces) is balanced by viscous drag from both the condensate and the tethers. Here, the drag is controlled by the mobility coefficients $M_{\phi }$ and $M_{\psi }$. Thus, the droplet velocity is given by

(7) $$-\frac{\partial E}{\partial x}=\gamma_{\text{drag}}\dot{x}=\left(\gamma_{\phi }M_{\phi }^{-1}+\gamma_{\psi }M_{\psi }^{-1}\right)\dot{x},$$

where the driving force $F_{\text{drive}}=-\partial_{x}E(x)$ is along the long axis of the tubule, where E(x) is the total energy for the droplet at position x. The precise form of E(x) is not needed for the analysis below, although it can be estimated by E(V,r), which is the energy of a droplet of volume V wetting a tubule of local radius r(x) (see Sec. III A of the Supplemental Material [18]). $\gamma_{\phi }M_{\phi }^{-1}$ and $\gamma_{\psi }M_{\psi }^{-1}$ represent the drag arising from diffusive transport of the condensate and the tethers, respectively (see Sec. III B of the Supplemental Material [18] for detailed derivation). Thus, droplet speed depends on tether mobility via an inverse linear relationship, $|\dot{x}|=\left(f_{\phi }+\right.{\left.f_{\psi }/M_{\psi }\right)}^{-1}$, with $f_{\phi }=\gamma_{\phi }M_{\phi }^{-1}/\left|\partial_{x}E\right|$ and $f_{\psi }=\gamma_{\psi }/\left|\partial_{x}E\right|$. This relation between $\left|\dot{x}\right|$ and $M_{\psi }$ is in good agreement with numerical simulations [Fig. 3(e), with $f_{\psi }$ and $f_{\phi }$ as fitting parameters]. The ratio of coefficients $f_{\psi }/f_{\phi }=M_{\phi }\gamma_{\psi }/\gamma_{\phi }$ depends on tether concentrations $\psi_{\text{dil}}$ and $\psi_{\text{den}}$. We estimate this ratio to be proportional to $\psi_{\text{dil}}\left(\psi_{\text{den}}-\psi_{\text{dil}}\right)$, which is confirmed numerically by fitting $\left|\dot{x}\right|$ to obtain $f_{\psi }$ and $f_{\phi }$ (see Sec. III B of the Supplemental Material [18] for details). In other words, tethers can slow down droplet migration if they cannot redistribute quickly enough to maintain an energetically favorable wetting configuration as the droplet moves. In the limit of immobile tethers $\left(M_{\psi }\to 0\right)$, the droplet becomes trapped in place.

Taken together, our results show that mobile tethers provide a mechanism to control how condensates respond to membrane geometry by modulating both the condensate’s favorable location and its migration speed.

## III. DISCUSSION

Membrane proteins and specialized lipids play an important role in regulating membrane functions, including their interaction with biomolecular condensates. However, the mobility of tethering molecules within the membrane has been largely overlooked in previous studies of condensate wetting. Here, we develop a general theoretical framework for mobile-tether-mediated wetting and show that tethering molecules can substantially modulate both equilibrium and dynamical aspects of condensate wetting, including migration and localization. These results suggest potential mechanisms for cells to regulate condensate formation and organization via the expression of mobile tethering molecules.

Our theory is relevant for a wide range of biological systems, including the algal pyrenoid [13,14,36], focal adhesion proteins [37,38], T-cell activation [39], actin assembly [40], and potentially the organization of ER exit sites [41–43]. Since a modest tether density and per-tether binding energy (a few $k_{\text{B}}T$) would be sufficient to substantially affect wetting properties, it is plausible for cells to regulate a wide range of condensates via mobile tethering molecules. Experimentally perturbing tethering molecules in cells will provide valuable insights into their importance for these structures.

Besides providing insights into *in vivo* structures and functions, our framework also makes quantitative predictions that can be tested *in vitro*. One direct test would be to place fluorescently tagged tethering molecules in supported lipid bilayers and track the tether concentrations $\psi_{\text{dil}}$ and $\psi_{\text{den}}$ as the membrane is wetted by a condensate that is attracted to the tether molecule. Repeating such experiments at different tether concentrations $\psi_{\text{dil}}$ would enable a quantitative test for the tether enrichment predicted by theory [Eq. (4) and Fig. 1(c)]. In addition, the contact angle can potentially be measured by confocal imaging and compared with theory [Eq. (5) and Fig. 1(d)].

While this work focuses on tether-mediated wetting of a fixed membrane, our framework can be extended to include effects such as membrane deformation [44–46] and active processes [47], such as post-translational modification upon wetting. An important extension is to take hydrodynamic coupling into account [48–50], for instance, by using model H [23] to describe condensate and tether dynamics. Another potential direction is to study the role of tethers in the prewetting regime, where a thin surface phase could emerge at concentrations below the binodal for bulk phase separation [51–54]. Going beyond deterministic field theories, it will be interesting to study how mobile tethers affect rare events such as nucleation [55]. In a biological context, it will also be interesting to study how tether-mediated wetting affects downstream signaling, which is often a nonequilibrium process [39, 40, 56–58]. It may also be rewarding to explore how tethers affect the way condensates navigate membrane structures with complex geometries, such as endoplasmic reticulum (connected sheets), mitochondria (cristae), and endosomes (tubulovesicular structures). Overall, our framework paves the way for the study of how mobile-tether-mediated interactions affect condensate morphology, dynamics, and function.

## Supplementary Material

SI movie 1 M1.0

SI movie 2 MO.1

Supplement

## Data Availability

The data that support the findings of this article are openly available [59].
