# Supplementary material for: Membrane-Anchored Mobile Tethers Modulate Condensate Wetting, Localization, and Migration
Source: PRX Life. Author manuscript; Available in PMC 2026 Jun 12. (PMC13256346; doi:10.1103/kxpb-9srd)
Supplement: Supplement [file NIHMS2178135-supplement-Supplement.pdf]

Supplemental Material: Membrane-anchored mobile tethers modulate condensate wetting, localization, and migration

Qiwei Yu,[1,*] Trevor GrandPre,[1,2,3,*] Andrew G.T. Pyo,[2,4] Andrej Košmrlj,[5,6,†] and Ned S. Wingreen[1,7,‡]

[1] *Lewis-Sigler Institute for Integrative Genomics, Princeton University, Princeton, NJ 08544*
[2] *Department of Physics, Princeton University, Princeton, NJ 08544*
[3] *Department of Physics and Center for Biomolecular Condensates, Washington University in St. Louis, St. Louis, MO 63130*
[4] *Department of Applied Physics, Stanford University, Stanford, CA 94305*
[5] *Department of Mechanical and Aerospace Engineering, Princeton University, Princeton, NJ 08544*
[6] *Princeton Materials Institute, Princeton University, Princeton, NJ 08544*
[7] *Department of Molecular Biology, Princeton University, Princeton, NJ 08544*

CONTENTS

I. Theoretical framework S1
A. Model S1
B. Derivation for tether-mediated enrichment and wetting S3
C. Generality of the results: Additional numerical simulations S5
1. Allowing $\chi_\psi \neq 0$ for tethers S5
2. Varying the tether-condensate interaction strength h_1. S6
3. Varying the form of the free energy S6

II. Estimating the tether binding energy required to drive wetting transition S6

III. Droplet migration on a tubule of varying radius S7
A. Equilibrium position of a droplet S7
B. Droplet migration velocity S8

IV. Details of numerical simulations S10

References S11

I. THEORETICAL FRAMEWORK

A. Model

As discussed in the main text, we consider a system where (three-dimensional) biomolecular condensates can interact with a (two-dimensional) membrane. The total free energy reads:

$$\beta F = c_{\psi,0} \int \mathrm{d}A \left[f_\psi(\psi) + \frac{\lambda_\psi}{2} (\nabla\psi)^2 - E(\psi, \phi|_{\mathrm{surf}}) \right] + c_{\phi,0} \int \mathrm{d}V \left[f_\phi(\phi) + \frac{\lambda_\phi}{2} (\nabla\phi)^2 \right], \tag{S1}$$

where ϕ is the condensate density field and ψ is the tether density field. The first integral is over the membrane area, while the second integral is over the bulk volume. $f_\phi(\phi)$ and $f_\psi(\psi)$ are the free-energy densities of the condensate and tethers, respectively; $E(\psi, \phi|_{\mathrm{surf}})$ describes the interaction energy between the condensate and the tether/membrane, with $\phi|_{\mathrm{surf}}$ denoting the condensate density at the membrane surface. λ_ψ and λ_ϕ are related to the line/surface tensions. The free energy is measured in units of $\beta^{-1} = k_{\mathrm{B}}T$. While the model can be used to describe a large class of systems by allowing the free-energy densities $f_\phi(\phi)$ and $f_\psi(\psi)$ and the interaction energy $E(\psi, \phi|_{\mathrm{surf}})$ to take different forms, we will focus on a minimal model to illustrate the essential physical picture.

---

* These authors contributed equally to this work.
† andrej@princeton.edu
‡ wingreen@princeton.edu

The tether and condensate fields evolve following gradient (model-B) dynamics that minimize the free energy [1]:

$$\partial_t \psi = \nabla \cdot (M_\psi \nabla \mu_\psi), \quad \partial_t \phi = \nabla \cdot (M_\phi \nabla \mu_\phi), \tag{S2}$$

where M_ψ and M_ϕ are mobility coefficients, while $\mu_\psi = \delta F/\delta \psi$ and $\mu_\phi = \delta F/\delta \phi$ are the chemical potentials of the tethers and condensate, respectively. The condensate obeys no-flux boundary condition at the membrane surface:

$$\hat{n} \cdot \nabla \mu_\phi = 0, \tag{S3}$$

where $\hat{n}$ is the unit normal vector pointing out of the membrane. Additionally, the bulk-surface interaction gives the following wetting boundary condition:

$$\lambda_\phi \hat{n} \cdot \nabla \phi|_{\mathrm{surf}} = -\partial_\phi E(\psi, \phi|_{\mathrm{surf}}) \cdot \frac{c_{\psi,0}}{c_{\phi,0}}, \tag{S4}$$

which reflects a local change in condensate concentration near the membrane surface due to the condensate's interaction with the membrane and tethers. Briefly, this can be derived by taking the variation of the total free energy and collecting terms that involve the condensate concentration at the surface, $\phi|_{\mathrm{surf}}$:

$$\beta \delta F \sim -c_{\psi,0} \int \mathrm{d}A \, \partial_\phi E(\psi, \phi|_{\mathrm{surf}}) \delta \phi|_{\mathrm{surf}} + c_{\phi,0} \int \mathrm{d}V \left[f'_\phi(\phi) \delta \phi + \lambda_\phi \nabla \phi \cdot \nabla \delta \phi \right] + \cdots, \tag{S5}$$

$$\sim -c_{\psi,0} \int \mathrm{d}A \, \partial_\phi E(\psi, \phi|_{\mathrm{surf}}) \delta \phi|_{\mathrm{surf}} - c_{\phi,0} \int \mathrm{d}A \, \lambda_\phi \hat{n} \cdot \nabla \phi|_{\mathrm{surf}} \delta \phi|_{\mathrm{surf}} + \cdots, \tag{S6}$$

where $\cdots$ contains terms that do not involve $\delta\phi|_{\mathrm{surf}}$. The second line is obtained by integrating the $\nabla\phi \cdot \nabla\delta\phi$ term by parts and lumping the volume integral term into $\cdots$ since they do not involve $\delta\phi|_{\mathrm{surf}}$. The minus sign is because the normal vector $\hat{n}$ is defined to point out of the membrane/into the condensate. Setting the coefficient of $\delta\phi|_{\mathrm{surf}}$ to zero gives the wetting boundary condition Eq. (S4).

Without loss of generality, we perform the following non-dimensionalization: length is rescaled by l_0, time is rescaled by t_0, and energy is rescaled by E_0. The non-dimensionalized free energy reads:

$$\tilde{F} = \frac{F}{E_0} = \frac{k_{\mathrm{B}} T l_0^2 c_{\psi,0}}{E_0} \int \mathrm{d}\tilde{A} \left[f_\psi(\psi) + \frac{\tilde{\lambda}_\psi}{2} \left(\tilde{\nabla} \psi \right)^2 - E(\psi, \phi|_{\mathrm{surf}}) \right] + \frac{k_{\mathrm{B}} T l_0^3 c_{\phi,0}}{E_0} \int \mathrm{d}\tilde{V} \left[f_\phi(\phi) + \frac{\tilde{\lambda}_\phi}{2} (\tilde{\nabla} \phi)^2 \right], \tag{S7}$$

where $\tilde{\nabla} = \partial_{\tilde{x}} = l_0 \nabla$, $\tilde{\lambda}_* = \lambda_*/l_0^2$, with $* \in \{\phi, \psi\}$. f and E do not need to be rescaled because they already represent the per-concentration energy densities. The non-dimensionalized dynamical equations read:

$$\partial_{\tilde{t}} \psi = \tilde{\nabla} \cdot \left(\tilde{M}_\psi \tilde{\nabla} \tilde{\mu}_\psi \right), \quad \partial_{\tilde{t}} \phi = \tilde{\nabla} \cdot \left(\tilde{M}_\phi \tilde{\nabla} \tilde{\mu}_\phi \right), \tag{S8}$$

where $\tilde{M}_* = M_* t_0 E_0 / l_0^2$ with $* \in \{\phi, \psi\}$ is the non-dimensionalized mobility coefficient, and $\tilde{\mu}_* = \partial_* \tilde{F}$ is the non-dimensionalized chemical potential. The wetting boundary condition is also non-dimensionalized:

$$\tilde{\lambda}_\phi \hat{n} \cdot \tilde{\nabla} \phi|_{\mathrm{surf}} = -\partial_\phi E(\psi, \phi|_{\mathrm{surf}}) \cdot \frac{c_{\psi,0}}{c_{\phi,0} l_0}. \tag{S9}$$

Without loss of generality, we consider the following non-dimensionalization:

$$l_0 = c_{\psi,0}/c_{\phi,0}, \quad E_0 = c_{\phi,0} k_{\mathrm{B}} T l_0^3, \quad t_0 = l_0^2/(M_\phi E_0). \tag{S10}$$

The first two conditions remove the coefficients in total free energy: $\frac{k_{\mathrm{B}} T l_0^2 c_{\psi,0}}{E_0} = 1$ and $\frac{k_{\mathrm{B}} T l_0^3 c_{\phi,0}}{E_0} = 1$; the coefficient in the wetting boundary condition is also removed: $\frac{c_{\psi,0}}{c_{\phi,0} l_0} = 1$. The last condition sets the rescaled condensate mobility $\tilde{M}_\phi = 1$.

To summarize the non-dimensionalized system, omitting the ˜ notation for the sake of simplicity:

$$F = \int \mathrm{d}A \left[f_\psi(\psi) + \frac{\lambda_\psi}{2} (\nabla \psi)^2 - E(\psi, \phi|_{\mathrm{surf}}) \right] + \int \mathrm{d}V \left[f_\phi(\phi) + \frac{\lambda_\phi}{2} (\nabla \phi)^2 \right], \tag{S11}$$

$$\partial_t \psi = \nabla \cdot (M_\psi \nabla \mu_\psi), \quad \partial_t \phi = \nabla^2 \mu_\phi, \tag{S12}$$

$$\lambda_\phi \hat{n} \cdot \nabla \phi|_{\mathrm{surf}} = -\partial_\phi E(\psi, \phi|_{\mathrm{surf}}), \quad \hat{n} \cdot \nabla \mu_\phi = 0. \tag{S13}$$

Eqs. (S11), (S12), and (S13) are the non-dimensionalized system that we will study hereafter (and for most of the main text). The only place where we need to explicitly consider dimensionality is for estimating the energy scales for real tethers (see the last paragraph of Section II.B in the main text, and Section II in the Supplementary Material).

B. Derivation for tether-mediated enrichment and wetting

For the sake of simplicity, we use Flory-Huggins free-energy densities for both the condensate and the tethers

$$f_\psi(\psi) = \psi \ln \psi + (1-\psi)\ln(1-\psi) + \chi_\psi \psi(1-\psi), \tag{S14}$$

$$f_\phi(\phi) = \phi \ln \phi + (1-\phi)\ln(1-\phi) + \chi_\phi \phi(1-\phi), \tag{S15}$$

with ψ and ϕ denoting the area or volume fraction of tether and condensate, respectively. χ_ψ and χ_ϕ are the Flory-Huggins interaction parameters for the tether and the condensate, respectively.

We further assume a linear interaction energy (non-dimensionalized in the same way as f_ψ)

$$E(\psi,\phi) = (h_0 + h_1\psi)\phi, \tag{S16}$$

where h_0 and h_1 describe condensate-membrane and condensate-tether interactions, respectively. The chemical potentials are given by:

$$\mu_\psi = \frac{\delta F}{\delta \psi} = \ln\frac{\psi}{1-\psi} + \chi_\psi(1-2\psi) - \lambda_\psi \nabla^2\psi - h_1\phi|_{z=0}, \tag{S17}$$

$$\mu_\phi = \frac{\delta F}{\delta \phi} = \ln\frac{\phi}{1-\phi} + \chi_\phi(1-2\phi) - \lambda_\phi \nabla^2\phi, \tag{S18}$$

where z is the perpendicular distance from the membrane, with $z=0$ indicating the membrane surface.

a. Tether enrichment. Let $\phi_{\rm den}$ and $\phi_{\rm dil}$ denote condensate densities in the dense phase and the dilute phase, respectively. The corresponding tether densities in the dense and dilute phases are denoted as $\psi_{\rm den}$ and $\psi_{\rm dil}$. The tether chemical potential is balanced between these two homogeneous phases:

$$\mu_\psi(\psi_{\rm den}, \phi_{\rm den}) = \mu_\psi(\psi_{\rm dil}, \phi_{\rm dil}) \tag{S19}$$

$$\Rightarrow \quad \ln\frac{\psi_{\rm den}}{1-\psi_{\rm den}} - h_1\phi_{\rm den}|_{z=0} - 2\chi_\psi\psi_{\rm den} = \ln\frac{\psi_{\rm dil}}{1-\psi_{\rm dil}} - h_1\phi_{\rm dil}|_{z=0} - 2\chi_\psi\psi_{\rm dil}. \tag{S20}$$

Approximating the surface density $\phi|_{z=0}$ with the bulk binodal values[1], we have [Eq. (3) in the main text]:

$$\ln\frac{\psi_{\rm den}}{1-\psi_{\rm den}} = \ln\frac{\psi_{\rm dil}}{1-\psi_{\rm dil}} + h_1\Delta\phi + 2\chi_\psi(\psi_{\rm den} - \psi_{\rm dil}), \tag{S21}$$

where $\Delta\phi = \phi_{\rm den}|_{z=0} - \phi_{\rm dil}|_{z=0} \approx \phi_{\rm den} - \phi_{\rm dil}$ is the difference between binodal concentrations.

In the limit of purely entropic tethers ($\chi_\psi = 0$), $\psi_{\rm den}$ can be solved for analytically:

$$\psi_{\rm den} = \frac{\psi_{\rm dil} e^{h_1\Delta\phi}}{1 + \psi_{\rm dil}(e^{h_1\Delta\phi} - 1)}, \tag{S22}$$

which is Eq. (4) in the main text. For $\chi_\psi \neq 0$, $\psi_{\rm den}$ can be solved for numerically. These results describe tether enrichment in the condensate during its wetting.

b. Wetting properties. The contact angle θ is given by force balance at the three-phase junction:

$$\sigma_{\rm den,dil}\cos\theta = \sigma_{\rm mem,dil} - \sigma_{\rm mem,den} \equiv \Delta\sigma, \tag{S23}$$

where the σs represent surface tensions. $\sigma_{\rm den,dil}$ is the surface tension between the dense and dilute phases in 3D and is independent of the tether concentration. The surface tension with the membrane is $\sigma_{{\rm mem},*}$, with $* \in \{{\rm den}, {\rm dil}\}$ denoting the dense or dilute phase, respectively. They are given by computing the excess free energy per unit area:

$$\sigma_{{\rm mem},*} = f_\psi(\psi_*) - E(\psi_*, \phi_*|_{z=0}) - \mu_\psi(\psi_*, \phi_*|_{z=0})\psi_* + \Delta f_{\rm excess}(\psi_*, \phi_*) \equiv \tilde\sigma_{s*} + \Delta f_{\rm excess}(\psi_*, \phi_*), \tag{S24}$$

with $* \in \{{\rm den}, {\rm dil}\}$. $\mu_\psi(\psi_*, \phi_*|_{z=0}) = \ln\frac{\psi_*}{1-\psi_*} + \chi_\psi(1-2\psi_*) - h_1\phi_*|_{z=0}$ is the tether chemical potential. $\Delta f_{\rm excess}(\psi_*, \phi_*)$ is defined below and will prove to be higher order in h_0 and h_1. We define $\tilde\sigma_{{\rm mem},*}$ to be the sum of the first three

[1] The correction due to this approximation is $\mathcal{O}(h_0^2, h_1^2)$, which, as shown below, is a higher-order term.

terms, which is given by

$$\begin{aligned}\tilde{\sigma}_{\mathrm{mem},*} =& f_\psi(\psi_*) - E(\psi_*, \phi_*|_{z=0}) - \mu_\psi(\psi_*, \phi_*|_{z=0})\psi_* \\ =&\psi_* \ln\psi_* + (1-\psi_*)\ln(1-\psi_*) + \chi_\psi\psi_*(1-\psi_*) - (h_0 + h_1\psi_*)\phi_* - \psi_*\left(\ln\frac{\psi_*}{1-\psi_*} + \chi_\psi(1-2\psi_*) - h_1\phi_*\right) \\ =& \ln(1-\psi_*) + \chi_\psi\psi_*^2 - h_0\phi_*. \end{aligned} \tag{S25}$$

The surface tension difference due to these terms reads

$$\Delta\tilde{\sigma} \equiv \tilde{\sigma}_{\mathrm{mem,dil}} - \tilde{\sigma}_{\mathrm{mem,den}} = h_0(\phi_{\mathrm{den}} - \phi_{\mathrm{dil}}) + \ln\frac{1-\psi_{\mathrm{dil}}}{1-\psi_{\mathrm{den}}} - \chi_\psi(\psi_{\mathrm{den}}^2 - \psi_{\mathrm{dil}}^2) \equiv \Delta\sigma_0 + \Delta\sigma_1, \tag{S26}$$

where $\Delta\sigma_0 = h_0(\phi_{\mathrm{den}} - \phi_{\mathrm{dil}}) = h_0\Delta\phi$ is the surface tension difference in the absence of tethers, and $\Delta\sigma_1 = \ln\frac{1-\psi_{\mathrm{dil}}}{1-\psi_{\mathrm{den}}} - \chi_\psi(\psi_{\mathrm{den}}^2 - \psi_{\mathrm{dil}}^2)$ is the additional surface tension difference due to mobile tethers. Note that to the leading order, we have $\Delta\tilde{\sigma} = \mathcal{O}(h_0, h_1)$.

$\Delta f_{\mathrm{excess}}(\psi_*, \phi_*|_{z=0})$ is the excess free-energy density due to a boundary layer of condensate at the membrane surface. Let $\hat{\phi}_*(z)$ denote the condensate concentration at distance z from the membrane in the dense ($*=$ den) and dilute ($*=$ dil) phases. Near the membrane, $\hat{\phi}_*(z)$ deviate from the binodal concentrations ϕ_*, leading to excess free energy per surface area:

$$\Delta f_{\mathrm{excess}}(\psi_*, \phi_*) = \int \mathrm{d}z_1 \left[g_\phi(\hat{\phi}_*(z_1)) + \frac{\lambda_\phi}{2}(\partial_z\hat{\phi}_*(z_1))^2 - g_\phi(\phi_*)\right], \tag{S27}$$

where ϕ_* denotes the binodal concentration, and $g_\phi(\phi) = f_\phi(\phi) - \mu_\phi\phi$ is the Gibbs free energy of the condensate. The concentration profile $\hat{\phi}_*(z_1)$ is the solution to the following boundary value problem:

$$\partial_z\mu_{\hat{\phi}}(z) = 0, \quad z > 0, \tag{S28}$$

$$\lambda_\phi\partial_z\hat{\phi}_*(z) = -(h_0 + h_1\psi_*), \quad z = 0, \tag{S29}$$

$$\lim_{z\to\infty}\hat{\phi}_*(z) = \phi_*. \tag{S30}$$

In practice, we find that the excess free energy density is negligible compared to the other terms. This can be explained by the following scaling argument: To the leading order in $\delta\phi_*(z) = \hat{\phi}_*(z) - \phi_*$, the excess free energy is

$$\Delta f_{\mathrm{excess}} \sim \frac{\Delta z}{2}\cdot\left[\lambda_\phi\delta\phi_*'(z)^2 + g''(\phi)\delta\phi_*(z)^2\right] = \mathcal{O}(\delta\phi_*^2), \tag{S31}$$

where Δz is the boundary layer thickness, and we have used $g'(\phi) = 0$ at the binodal concentration. From the wetting condition [Eq. (S29)], we find $\delta\phi_* = \mathcal{O}(h_0, h_1)$. Hence, the excess free energy becomes quadratic in the interaction parameters:

$$\Delta f_{\mathrm{excess}} = \mathcal{O}\big(h_0^2, h_1^2\big), \tag{S32}$$

which is a higher-order term compared to $\Delta\tilde{\sigma}$.

In summary, to the leading order of $\mathcal{O}(h_0, h_1)$, the contact angle is given by

$$\cos\theta = \frac{\sigma_{\mathrm{mem,dil}} - \sigma_{\mathrm{mem,den}}}{\sigma_{\mathrm{den,dil}}} \approx \frac{\Delta\tilde{\sigma}}{\sigma_{\mathrm{den,dil}}} = \frac{\Delta\sigma_0 + \Delta\sigma_1}{\sigma_{\mathrm{den,dil}}}, \tag{S33}$$

where $\Delta\sigma_0 = h_0(\phi_{\mathrm{den}} - \phi_{\mathrm{dil}}) = h_0\Delta\phi$ is the surface tension difference in the absence of tethers, and $\Delta\sigma_1 = \ln\frac{1-\psi_{\mathrm{dil}}}{1-\psi_{\mathrm{den}}} - \chi_\psi(\psi_{\mathrm{den}}^2 - \psi_{\mathrm{dil}}^2)$ is the additional surface tension difference due to mobile tethers. This produces Eq. (5) in the main text.

c. Wetting with purely entropic tethers. In the limit of purely entropic tethers ($\chi_\psi = 0$), we can substitute ψ_{den} with Eq. (S22) to get a simplified expression for $\Delta\sigma_1$:

$$\Delta\sigma_1 = \ln\left[1 + \psi_{\mathrm{dil}}(e^{h_1\Delta\phi} - 1)\right], \tag{S34}$$

which produces Eq. (6) in the main text. The contact angle simplifies to

$$\cos\theta = \frac{h_0(\phi_{\mathrm{den}} - \phi_{\mathrm{dil}}) + \ln\left[1 + \psi_{\mathrm{dil}}(e^{h_1\Delta\phi} - 1)\right]}{\sigma_{\mathrm{den,dil}}}. \tag{S35}$$

To achieve complete wetting ($\cos\theta = 1$), the critical tether density $\psi_{\mathrm{dil}}^\star$ is given by

$$\sigma_{\mathrm{den,dil}} = h_0\Delta\phi + \ln\left[1 + \psi_{\mathrm{dil}}^\star(e^{h_1\Delta\phi} - 1)\right] \Rightarrow \psi_{\mathrm{dil}}^\star = \frac{e^{\sigma_{\mathrm{den,dil}} - h_0\Delta\phi} - 1}{e^{h_1\Delta\phi} - 1}. \tag{S36}$$

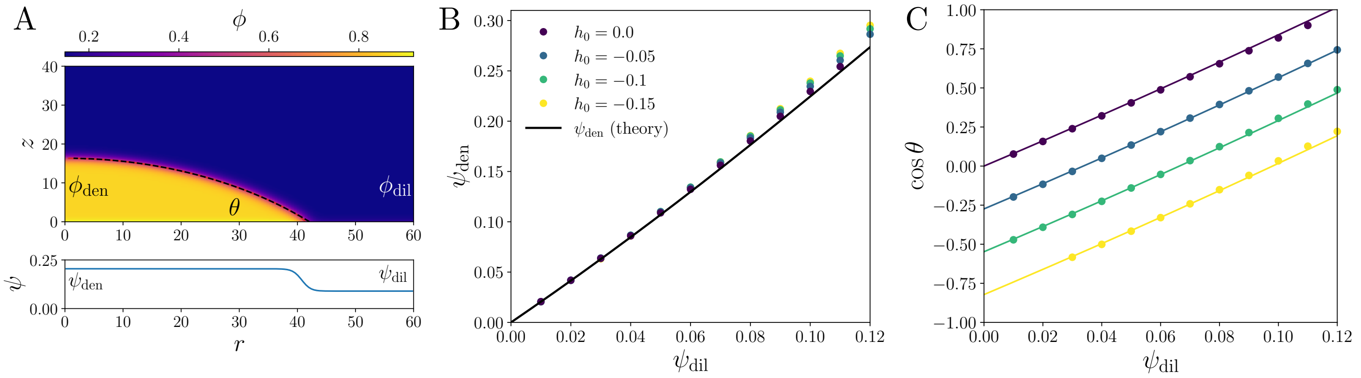

FIG. S1. Condensate wetting of membranes mediated by mobile tethers with $\chi_\psi = 1$ and $\lambda_\psi = 1$; all the other parameters are identical to Fig. 1 in the main text. (A) A typical equilibrium concentration profile obtained from numerical simulations. The condensate field ϕ (top) and tether field ψ (bottom) are plotted in cylindrical coordinates (r, z) with axial symmetry. The membrane is flat at $z = 0$. The black dashed curve is a spherical cap fit to the condensate surface contour. Parameters: $h_0 = 0$, $\psi_{\rm dil} = 0.09$. (B) Condensate-enriched tether concentration $\psi_{\rm den}$ increases with bulk tether concentration $\psi_{\rm dil}$, for different condensate-membrane interactions h_0, consistent with theory [solid curve, by numerically solving Eq. (S21) for $\psi_{\rm den}$]. (C) Contact angle $\cos\theta$ as a function of tether concentration $\psi_{\rm dil}$ for different h_0 (see legend in B) agrees well with theory [solid curves, Eq. (S33)].

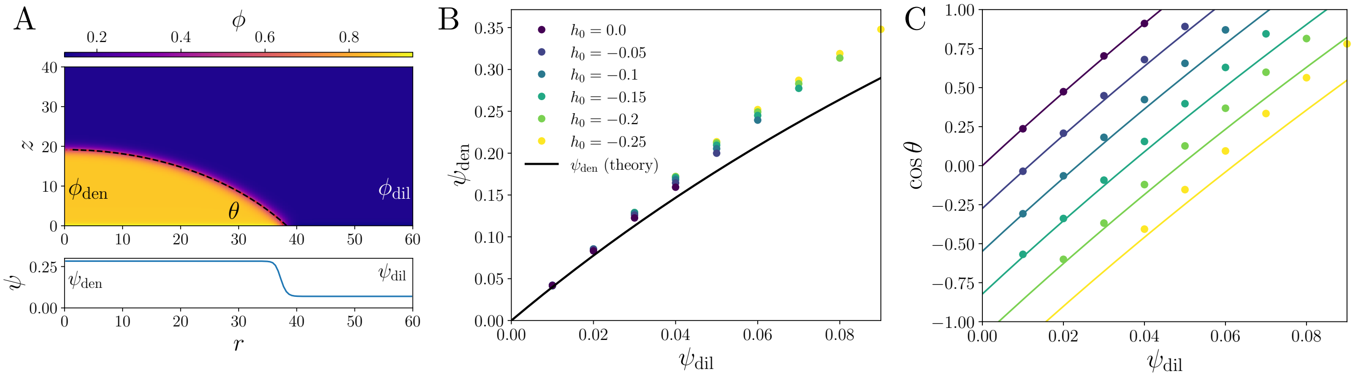

FIG. S2. Tether-mediated condensate wetting with a stronger tether-condensate interaction strength ($h_1 = 2.0$; all the other parameters are identical to Fig. 1 in the main text). (A) A typical equilibrium concentration profile obtained from numerical simulations (see Fig. S1A caption for a detailed description) for $h_0 = -0.2$ and $\psi_{\rm dil} = 0.07$. (B) Condensate-enriched tether concentration $\psi_{\rm den}$ increases with bulk tether concentration $\psi_{\rm dil}$, for different condensate-membrane interactions h_0, compared with theory [solid curve, Eq. (S22)]. (C) Contact angle $\cos\theta$ as a function of tether concentration $\psi_{\rm dil}$ for different h_0 (see legend in B) agrees well with theory [solid curves, Eq. (S35)]. Note that since the theory is only to the leading order $\mathcal{O}(h_0, h_1)$, it becomes less accurate at large h and $\psi_{\rm dil}$.

C. Generality of the results: Additional numerical simulations

Fig. 1 in the main text demonstrates good agreement between theory and numerical simulation of the full model. To illustrate the physical picture, it focuses on the simplest case of Flory-Huggins free energy, purely entropic tethers ($\chi_\psi = 0$ and $\lambda_\psi = 0$), and tether-condensate interaction strength $h_1 = 1$. Here, we demonstrate that our results are general and not sensitive to the specific choices of parameters or functional forms of the free energy.

1. *Allowing $\chi_\psi \neq 0$ for tethers*

In the main text, we focus on the case of purely entropic tethers ($\chi_\psi = 0$) to take advantage of analytical tractability [Eqs. (S22) and (S35)] to illustrate the physical picture. However, our analysis is also valid for $\chi_\psi \neq 0$, which describes an energetic cost of mixing in the membrane. The only difference is that the tether enrichment and contact angle need to be determined numerically from Eqs. (S21) and (S33), respectively. Fig. S1 shows the comparison for $\chi_\psi = 1.0$

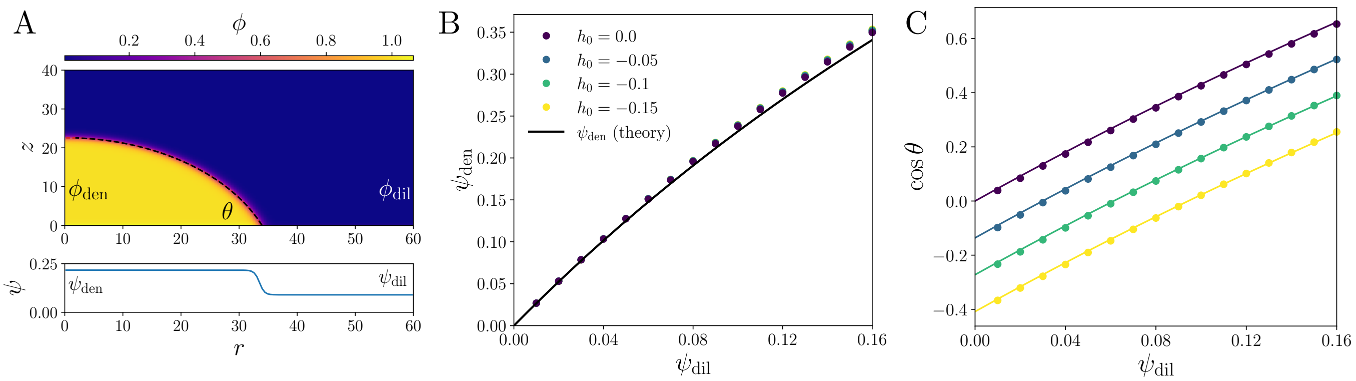

FIG. S3. Tether-mediated condensate wetting with a ϕ^4 free energy density for the condensate [Eq. (S37)]. $\chi_\psi = 2.5$ and all the other parameters are identical to Fig. 1 in the main text. (A) A typical equilibrium concentration profile obtained from numerical simulations (see Fig. S1A caption for a detailed description) for $h_0 = 0$ and $\psi_{\rm dil} = 0.09$. (B) Condensate-enriched tether concentration $\psi_{\rm den}$ increases with bulk tether concentration $\psi_{\rm dil}$, for different condensate-membrane interactions h_0, compared with theory [solid curve, Eq. (S22)]. (C) Contact angle $\cos\theta$ as a function of tether concentration $\psi_{\rm dil}$ for different h_0 (see legend in B) agrees well with theory [solid curves, Eq. (S35)].

and $\lambda_\psi = 1.0$. The theory accurately captures tether enrichment (Fig. S1B) and contact angle (Fig. S1C) for a wide range of h_0 and $\psi_{\rm dil}$.

2. *Varying the tether-condensate interaction strength h_1.*

As mentioned above, our main results [Eqs. (S22) and (S35)] are derived to the leading order of $\mathcal{O}(h_0, h_1)$. This allowed us to approximate the difference in surface concentration with the difference between binodal concentrations, i.e. $\Delta\phi = \phi_{\rm den}|_{z=0} - \phi_{\rm dil}|_{z=0} \approx \phi_{\rm den} - \phi_{\rm dil}$ and to neglect $\Delta f_{\rm excess}$ when computing the surface tension difference $\Delta\sigma$. As the tether-condensate interaction strength h_1 increases, we expect a thin boundary layer to form near the membrane where the condensate is enriched compared to the bulk, with the level of enrichment differing between dense and dilute phases. This effect leads to an $\mathcal{O}(h_0^2, h_1^2)$ correction to our theory.

Fig. S2 shows the comparison between theory and simulation for $h_1 = 2.0$. As expected, increasing h_1 did not change the qualitative behavior of $\psi_{\rm den}$ and $\cos\theta$, but the quantitative agreement is less satisfactory compared to Fig. 1 in the main text ($h_1 = 1.0$).

3. *Varying the form of the free energy*

The analytical results [Eqs. (S22) and (S35)] are general and do not require assuming any functional form for the condensate free energy $f_\phi(\phi)$, which was not used in the derivation in Section I.B. To demonstrate this, we use a ϕ^4 double-well free-energy density for the condensate:

$$f_\phi(\phi) = \chi_\phi \phi^2 (1-\phi)^2, \tag{S37}$$

with tethers still described by a Flory-Huggins free energy. Our analytical results [Eqs. (S22) and (S35)] still hold, with the binodal concentrations $\phi_{\rm dil}$ and $\phi_{\rm den}$ obtained from a Maxwell common tangent construction of $f_\phi(\phi)$. Fig. S3 shows excellent agreement between theory and simulation.

II. ESTIMATING THE TETHER BINDING ENERGY REQUIRED TO DRIVE WETTING TRANSITION

Here, we estimate the value of $\Delta\sigma_0$, which is the dilute/dense phase surface tension difference due to the condensate-membrane interaction. Typically, a membrane would be slightly repulsive for a polymer condensate because being close to the membrane reduces the conformational entropy of the polymers. Thus, we can estimate the magnitude of this effect by considering polymer "blobs" close to the membrane. Each "blob" would contribute $1 k_{\rm B}T$, and the number of

polymer "blobs" per unit area could be estimated by $1/R_g^2$ with R_g being the radius of gyration. For an intrinsically disordered protein (IDP) of length ~ 100 a.a., we estimate $R_g \sim 3$nm [2], and therefore $1/R_g^2 \sim \mathcal{O}(10^{-1})\text{nm}^2$. Thus, the surface tension due to entropic repulsion is of the order $\Delta\sigma_0 \sim -\mathcal{O}(10^{-1})k_\text{B}T/\text{nm}^2$.

On the other hand, previous micropipette aspiration found that the typical biopolymer condensate surface tension $\sigma_{\text{den,dil}}$ is at most $\mathcal{O}(1)$mN/m, or equivalently $\mathcal{O}(10^{-1})k_\text{B}T/\text{nm}^2$ [3]. Thus, to achieve complete wetting, the additional surface tension difference due to tethers must also reach $\Delta\sigma_1 = \sigma_{\text{den,dil}} - \Delta\sigma_0 \sim \mathcal{O}(10^{-1})k_\text{B}T/\text{nm}^2$.

To estimate the corresponding binding energy relevant for real tethers, we recall that during non-dimensionalization, the surface tension was renormalized by $\frac{E_0}{l_0^2} = c_{\psi,0}k_\text{B}T$, with a close-packed tether density set by $c_{\psi,0}$. Thus, in the limit of dilute tethers $\psi_{\text{dil}} = n/c_{\psi,0} \ll 1$, where n is the (dimensional) tether density in contact with the dilute phase, we have

$$\frac{\Delta\sigma_1}{k_\text{B}T} = c_{\psi,0}\ln\left[1+\psi_{\text{dil}}(e^{h_1\Delta\phi}-1)\right] \approx c_{\psi,0}\psi_{\text{dil}}(e^{h_1\Delta\phi}-1) = n(e^{\epsilon}-1), \tag{S38}$$

where $n \sim 10^{-2}\text{nm}^2$ [4] is the dimensional tether number density, and $\epsilon = h_1\Delta\phi$ is the energy reduction per tether when inside the condensate, measured in units of $k_\text{B}T$. This leads to

$$\epsilon = \ln\left(1+\frac{\Delta\sigma_1}{c_\text{g}k_\text{B}T}\right) \sim \ln\left[1+\mathcal{O}(10)\right] \sim \mathcal{O}(1)\ (k_\text{B}T), \tag{S39}$$

which suggests that a binding energy of a few $k_\text{B}T$ per tether is sufficient to modulate equilibrium condensate-membrane wetting properties.

III. DROPLET MIGRATION ON A TUBULE OF VARYING RADIUS

A. Equilibrium position of a droplet

In this section, we consider the free energy of a droplet as it wets a membrane tubule in an axisymmetric configuration that wraps around the tubule. If the radius of the tubule is slow-varying compared to the droplet size, we can approximate the tubule locally as a cylinder of radius r (Fig. S4A). Thus, the free energy of the droplet $E(V,r)$ is determined by its volume V and the local tubule radius r.

The droplet adopts a barrel-shaped constant-mean-curvature surface when wetting the cylinder [5]. For analytical tractability and motivated by our numerical simulations (Fig. S4B), we approximate the cross-section profile as a circular cap (Fig. S4A). The radius R is determined by constraining the volume of the droplet:

$$V(R,r,\cos\theta) = \frac{1}{6}\pi R^2(12\theta r - 6r\sin 2\theta + 9R\sin\theta + R\sin 3\theta - 12\theta R\cos\theta), \tag{S40}$$

where θ is the contact angle. The total surface free energy is given by

$$\begin{aligned} E(R,r,\cos\theta) &= A_{\text{mem,den}}\sigma_{\text{mem,den}} + A_{\text{mem,dil}}\sigma_{\text{mem,dil}} + A_{\text{den,dil}}\sigma_{\text{den,dil}} && \text{(S41)}\\ &= (A_0 - A_{\text{mem,den}})\sigma_{\text{mem,dil}} + A_{\text{mem,den}}\sigma_{\text{mem,den}} + A_{\text{den,dil}}\sigma_{\text{den,dil}} && \text{(S42)}\\ &= E_0 + A_{\text{mem,den}}(\sigma_{\text{mem,den}} - \sigma_{\text{mem,dil}}) + A_{\text{den,dil}}\sigma_{\text{den,dil}} && \text{(S43)}\\ &= E_0 + \sigma_{\text{den,dil}}(A_{\text{den,dil}} - A_{\text{mem,den}}\cos\theta), && \text{(S44)} \end{aligned}$$

where A_0 is the total membrane area, $E_0 = A_0\sigma_{\text{mem,dil}}$, and $A_{\text{mem,den}}$, $A_{\text{mem,dil}}$, and $A_{\text{den,dil}}$ are the areas of the membrane-condensate, membrane-solvent, and condensate-solvent interfaces, respectively. The last line follows from the force balance at the three-phase junction [Eq. (S23)]. Applying our droplet shape ansatz, we have

$$E(R,r,\cos\theta) = 4\pi R(\theta r - \cos\theta(r\sin\theta + \theta R) + R\sin\theta). \tag{S45}$$

The equilibrium position of the droplet is determined by the r_{eq} that minimizes the free energy $E(V,r)$ for a fixed droplet volume V. The constrained optimization problem can be solved by introducing a Lagrange multiplier λ:

$$\mathcal{L}(R,r,\cos\theta,\lambda) = E(R,r,\cos\theta) - \lambda(V(R,r,\cos\theta) - V_0). \tag{S46}$$

The optimum is given by

$$\frac{\partial\mathcal{L}}{\partial R} = 0, \quad \frac{\partial\mathcal{L}}{\partial r} = 0, \quad V(R,r,\cos\theta) = V_0. \tag{S47}$$

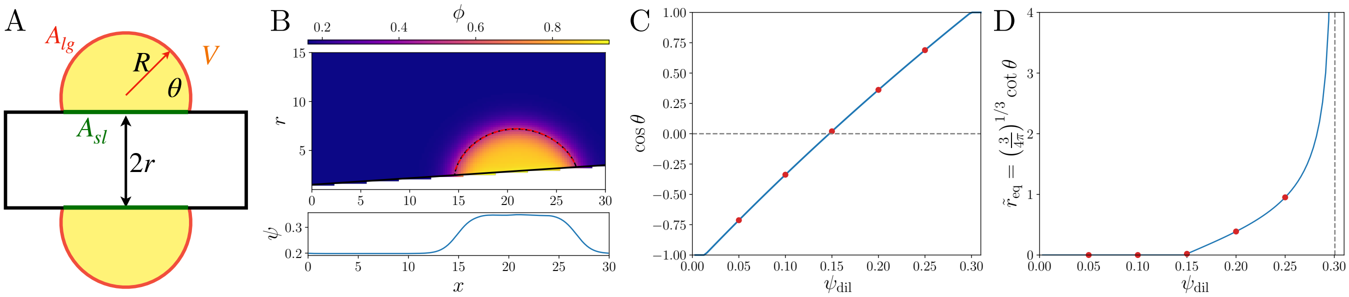

FIG. S4. Equilibrium position of a droplet on a tubule of varying radius. (A) Illustration of the ansatz used for computing the free energy of a droplet of volume V wetting a cylinder of radius r with contact angle θ. This is a cross-sectional view where the droplet assumes a circular cap shape with radius R. (B) Snapshot of the condensate profile ϕ (top) and tether profile ψ (bottom) for a droplet migrating on a tubule (membrane indicated by the black line) obtained from numerical simulation. The red dashed curve shows that a circular fit is in good agreement with the interface contour (black dashed curve). (C) Contact angle $\cos\theta$ as a function of tether density $\psi_{\rm dil}$. (D) Normalized equilibrium tubule radius $\tilde{r}_{\rm eq} \equiv r_{\rm eq}/V^{1/3}$ as a function of tether density $\psi_{\rm dil}$. In (C) and (D), blue curves are theoretical predictions [Eq. (S33)] and [Eq. (S49)]), and red data points simply indicate the theoretical predictions evaluated at the $\psi_{\rm dil}$ values used in Fig. 3 in the main text. In (D), the vertical dashed line indicates $\tilde{r}_{\rm eq} \to +\infty$ as $\theta \to 0$. Parameters: $h_0 = -0.2$, $h_1 = 1$, $\chi_\phi = 2.5$, $\lambda_\phi = 1$, $\chi_\psi = 0$, $\lambda_\psi = 0$. For (B), $\psi_{\rm dil} = 0.20$ and mobilities $M_\phi = 1.0$ and $M_\psi = 1.0$.

The solutions are:

$$\tilde{r} \equiv r/V^{1/3} = \left(\frac{3}{4\pi}\right)^{1/3} \cot\theta, \quad \tilde{R} \equiv R/V^{1/3} = \tilde{r}/\cos\theta = \left(\frac{3}{4\pi}\right)^{1/3} \csc\theta, \quad \lambda = \frac{2}{R}. \tag{S48}$$

Since the radius r must be positive, this solution is only physical for $\theta \in (0, \pi/2)$. For $\theta \in (\pi/2, \pi)$, the surface energy increases monotonically with r, and the droplet would prefer to reside at the smallest radius possible. Putting these results together, we arrive at the equilibrium radius of the tubule where the droplet prefers to locate:

$$r_{\rm eq} = \begin{cases} V^{1/3}\left(\frac{3}{4\pi}\right)^{1/3} \cot\theta, & \theta \in (0, \pi/2), \\ 0, & \theta \in (\pi/2, \pi). \end{cases} \tag{S49}$$

Hence, tuning tether abundance $\psi_{\rm dil}$ (and thus the contact angle θ) can control the equilibrium position of the droplet on a tubule of varying radius (Fig. S4C,D). Theoretical predictions for the $\psi_{\rm dil}$ values used in Fig. 3 in the main text are indicated by red data points: for $\psi_{\rm dil} = 0.05, 0.10$, the droplet moves to the left to decrease r; for $\psi_{\rm dil} = 0.15, 0.20, 0.25$, the droplet moves to the right to increase r.

B. Droplet migration velocity

As a droplet migrates on a tubule, its velocity $\dot{x}$ is determined by the balance between the driving force due to the surface energy gradient and the drag force:

$$F_{\rm drive} = -\frac{\partial E}{\partial x} = \gamma_{\rm drag}\dot{x} = (\gamma_\phi M_\phi^{-1} + \gamma_\psi M_\psi^{-1})\dot{x} = F_{\rm drag}, \tag{S50}$$

where $\gamma_{\rm drag}$ is the drag coefficient, which, as shown below, will be inversely proportional to the mobility coefficients M_ϕ and M_ψ of the condensate and tethers, respectively. γ_ϕ and γ_ψ are constants that depend on the concentration profile of the condensate and tethers, respectively, which will be derived below.

As derived in the previous section $E(V, r)$ depends on both the droplet volume V and the local tubule radius r, which gives the driving force $F_{\rm drive} = -\frac{\partial E}{\partial x} = -\frac{\partial E}{\partial r}\frac{\partial r}{\partial x}$. The velocity $\dot{x}$ depends on M_ψ via an inverse linear relation:

$$\dot{x} = \left(f_\phi + f_\psi M_\psi^{-1}\right)^{-1}, \tag{S51}$$

where

$$f_\phi = \frac{\gamma_\phi}{M_\phi} \cdot \left(-\frac{\partial E}{\partial r}\frac{\partial r}{\partial x}\right), \quad f_\psi = \gamma_\psi \cdot \left(-\frac{\partial E}{\partial r}\frac{\partial r}{\partial x}\right). \tag{S52}$$

Estimating f_ϕ and f_ψ requires computing the drag coefficients γ_ϕ and γ_ψ, which we turn to next.

The drag coefficient is determined from the total dissipation:

$$P = \gamma \dot{x}^2 = \int \mathrm{d}V \, \frac{\left|\vec{J}_\phi\right|^2}{M_\phi} + \int \mathrm{d}A \, \frac{\left|\vec{J}_\psi\right|^2}{M_\psi} = \int \mathrm{d}V \, M_\phi |\nabla \mu_\phi|^2 + \int \mathrm{d}A \, M_\psi |\nabla \mu_\psi|^2, \tag{S53}$$

where $\vec{J}_\phi = -M_\phi \nabla \mu_\phi$ and $\vec{J}_\psi = -M_\psi \nabla \mu_\psi$ are the fluxes of the condensate and tethers, respectively. Further integrating by parts, we have

$$P = -\int \mathrm{d}V \, M_\phi \mu_\phi \nabla^2 \mu_\phi - \int \mathrm{d}A \, M_\psi \mu_\psi \nabla^2 \mu_\psi. \tag{S54}$$

For a traveling wave solution, we have $\partial_t \phi = -\dot{x}\partial_x \phi$ and $\partial_t \psi = -\dot{x}\partial_x \psi$. Thus, their (model B) dynamics satisfy

$$\partial_t \psi = -\dot{x}\partial_x \psi = M_\psi \nabla^2 \mu_\psi, \quad \partial_t \phi = -\dot{x}\partial_x \phi = M_\phi \nabla^2 \mu_\phi. \tag{S55}$$

$\mu_{\phi,\psi}$ are solutions to Poisson equations with source terms $-\dot{x}\partial_x \phi / M_\phi$ and $-\dot{x}\partial_x \psi / M_\psi$, respectively. We can rescale the chemical potentials by the strength of the source:

$$\mu_\phi = \frac{\dot{x}}{M_\phi} \tilde{\mu}_\phi, \quad \mu_\psi = \frac{\dot{x}}{M_\psi} \tilde{\mu}_\psi, \tag{S56}$$

where $\tilde{\mu}_\phi$ and $\tilde{\mu}_\psi$ are solutions to the Poisson equations with unit source terms:

$$\nabla^2 \tilde{\mu}_\phi = -\partial_x \phi, \quad \nabla^2 \tilde{\mu}_\psi = -\partial_x \psi. \tag{S57}$$

Substituting these back into the expression for P, we have

$$P = -\int \mathrm{d}V \, \dot{x}^2 M_\phi^{-1} \tilde{\mu}_\phi \nabla^2 \tilde{\mu}_\phi - \int \mathrm{d}A \, \dot{x}^2 M_\psi^{-1} \tilde{\mu}_\psi \nabla^2 \tilde{\mu}_\psi, \tag{S58}$$

$$= \dot{x}^2 \left(\int \mathrm{d}V \, M_\phi^{-1} \tilde{\mu}_\phi \partial_x \phi + \int \mathrm{d}A \, M_\psi^{-1} \tilde{\mu}_\psi \partial_x \psi \right) = \gamma_{\mathrm{drag}} \dot{x}^2, \tag{S59}$$

which gives the coefficients

$$\gamma_\phi = \int \mathrm{d}V \, \tilde{\mu}_\phi \partial_x \phi = \int \mathrm{d}V \, (-\partial_x \tilde{\mu}_\phi) \phi, \tag{S60}$$

$$\gamma_\psi = \int \mathrm{d}A \, \tilde{\mu}_\psi \partial_x \psi = \int \mathrm{d}A \, (-\partial_x \tilde{\mu}_\psi) \psi. \tag{S61}$$

Since the chemical potential gradients $-\partial_x \tilde{\mu}_{\phi,\psi}$ are non-zero only at the interface region, we estimate that $\gamma_{\phi,\psi}$ are proportional to the interface area/length as well as to the concentrations:

$$\gamma_\phi \sim A_{\mathrm{den,dil}} \cdot \frac{\Delta\phi}{\lambda_\phi} \phi_{\mathrm{den}}, \quad \gamma_\psi \sim L_{\mathrm{mem,den,dil}} \cdot \frac{\Delta\psi}{\lambda_\psi} \psi_{\mathrm{den}}, \tag{S62}$$

where $\Delta\phi$ and $\Delta\psi$ are the concentration differences across the interface, λ_ϕ and λ_ψ are the interface thicknesses, $A_{\mathrm{den,dil}}$ is the area of the condensate-solvent interface, and $L_{\mathrm{mem,den,dil}}$ is the length of the three-phase contact line.

Assuming that the geometry and the condensate concentrations difference $\Delta\phi$ remain approximately unchanged with varying M_ψ (which is valid for the simulations in Fig. 3E), the main effect of changing tether abundance ψ_{dil} should be due to the ψ dependence in γ_ψ. Thus, we estimate:

$$\frac{f_\psi}{f_\phi} = M_\phi \frac{\gamma_\psi}{\gamma_\phi} \propto (\psi_{\mathrm{den}} - \psi_{\mathrm{dil}}) \psi_{\mathrm{dil}}. \tag{S63}$$

This is consistent with our numerical results (Fig. S5), where we find a linear relation between f_ψ / f_ϕ and $(\psi_{\mathrm{den}} - \psi_{\mathrm{dil}}) \psi_{\mathrm{dil}}$.

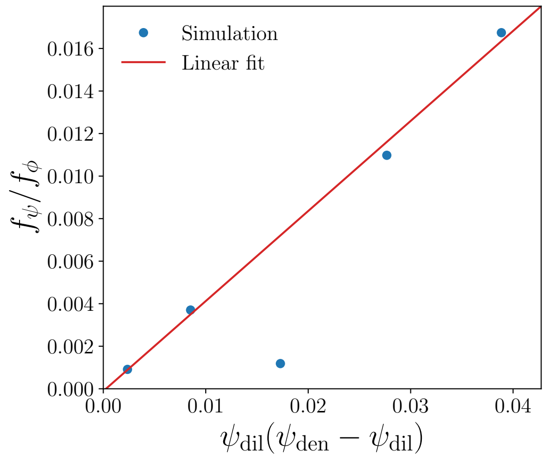

FIG. S5. Proportionality between f_ψ/f_ϕ and $(\psi_{\rm den}-\psi_{\rm dil})\psi_{\rm dil}$ [Eq. (S63)]. Blue circles are numerical results obtained from simulations in Fig. 3E, and the red line is a linear fit excluding the point at $\psi_{\rm dil}=0.15$, for which the droplet velocity is too small for an accurate estimate of the drag coefficients.

IV. DETAILS OF NUMERICAL SIMULATIONS

To solve the governing equations [Eqs. (S11), (S12), and (S13)] for the condensate and tethers, we use a finite-volume numerical scheme [6] with first-order forward Euler time-stepping for time evolution. The condensate field ϕ obeys no-flux boundary conditions at all boundaries in addition to wetting boundary conditions at membrane interfaces. The tether field ψ obeys Dirichlet boundary conditions with fixed bulk tether concentration $\psi_{\rm dil}$. The wetting boundary condition is prescribed using the ghost point method, where the ϕ value at each ghost point is interpolated from the two nearest interior points that are not collinear with the ghost point. The volumes near the boundary are treated with the cut-cell method. The implementation of cut-cell and ghost point methods was simplified from previous work [7–9].

For Fig. 1 (and Figs. S1, S2, and S3), the simulation is performed in cylindrical coordinates, with spatial discretization $r_n=\frac{nr_{\rm max}}{N}$, $z_m=\frac{mz_{\rm max}}{M}$, where $r_{\rm max}=60$ and $z_{\rm max}=40$ set the system size and $N=M=128$ set the spatial resolution. The simulation is run until the system reaches its equilibrium state. The contact angle is measured by fitting the contour to a spherical cap $R^2=r^2+(z-z_0)^2$, which gives $\cos\theta=-z_0/R_0$. $\sigma_{\rm den,dil}$ is evaluated from the phase field simulations by integrating the excess free energy density across a flat interface. The parameters for Fig. 1 are: $\chi_\phi=2.5$, $\lambda_\phi=1$, $\chi_\psi=0$, $\lambda_\psi=0$; $h_1=1$ for (B)–(D); $\psi_{\rm dil}=0.02$ and $h_0=0$ for (B); $h_0=-0.2$ for (E).

For Fig. 2, the simulation was performed in 2D planar coordinates, with spatial discretization $x_n=\frac{nx_{\rm max}}{N}$, $y_m=\frac{my_{\rm max}}{M}$ with $x_{\rm max}=y_{\rm max}=30$ and $N=M=64$. The parameters are: $h_0=-0.2$, $h_1=2$, $\chi_\phi=2.5$, $\lambda_\phi=1$, $\chi_\psi=0$, $\lambda_\psi=0$. Tether concentration is fixed at the boundary by Dirichlet boundary condition $\psi_{\rm dil}=0.05$. The videos for the dynamics are available as supplemental videos (`si_movie_1_M1.0.mp4` for $M_\psi=1.0$ and `si_movie_2_M0.1.mp4` for $M_\psi=0.1$).

To quantify how fast the droplet reaches its equilibrium configuration at the lower-left corner where the two membranes meet, we defined an average distance $\langle r\rangle=\int\delta\phi(x,y)\sqrt{x^2+y^2}\,{\rm d}x\,{\rm d}y\,/\int\delta\phi(x,y)\,{\rm d}x\,{\rm d}y$, where $\delta\phi=\phi-\phi_g$ is the condensate concentration subtracted by the dilute phase.

For Fig. 3 (and Figs. S4 and S5), the simulation was performed in cylindrical coordinates (r,x), where the x-axis runs along the center of the tubule. The tubule radius is given by $R(x)=R_0+R_1x$, with $R_0=1.5$ and $R_1=1/15$. Spatial discretization is given by $r_n=r_0+\frac{nr_{\rm max}}{N}$, $x_m=\frac{mx_{\rm max}}{M}$, where $r_0=1.0$, $r_{\rm max}=15$ and $x_{\rm max}=30$ set the system size and $N=64$ and $M=128$ set the spatial resolution. The equations are only evolved in grid points inside the domain $[r>R(x)]$ with wetting boundary conditions implemented by ghost points. The average position $\langle x\rangle$ is computed from the volume inside the contour $\phi>(\phi_{\rm den}+\phi_{\rm dil})/2$. Tether concentration is fixed at the boundary by Dirichlet boundary condition at the right boundary $(x=x_{\rm max})$. The parameters are: $h_0=-0.2$, $h_1=1$, $\chi_\phi=2.5$, $\lambda_\phi=1$, $\chi_\psi=0$, $\lambda_\psi=0$. For Fig. 3A, $\psi_{\rm dil}=0.20$ and $M_\psi=1.0$; for Fig. 3B, $M_\psi=1.0$; for Fig. 3C, $\psi_{\rm dil}=0.10$.

The simulations are performed in Python, with codes available at `https://github.com/qiweiyuu/tether`.

REFERENCES

[1] P. C. Hohenberg and B. I. Halperin, Theory of dynamic critical phenomena, Rev. Mod. Phys. **49**, 435 (1977).

[2] H. Hofmann, A. Soranno, A. Borgia, K. Gast, D. Nettels, and B. Schuler, Polymer scaling laws of unfolded and intrinsically disordered proteins quantified with single-molecule spectroscopy, Proc. Natl. Acad. Sci. U. S. A. **109**, 16155 (2012).

[3] H. Wang, F. M. Kelley, D. Milovanovic, B. S. Schuster, and Z. Shi, Surface tension and viscosity of protein condensates quantified by micropipette aspiration, Biophysical Reports **1** (2021).

[4] D. N. Itzhak, S. Tyanova, J. Cox, and G. H. Borner, Global, quantitative and dynamic mapping of protein subcellular localization, eLife **5**, e16950 (2016).

[5] B. J. Carroll, The accurate measurement of contact angle, phase contact areas, drop volume, and laplace excess pressure in drop-on-fiber systems, J. Colloid Interface Sci. **57**, 488 (1976).

[6] R. J. Leveque, *Finite volume methods for hyperbolic problems* (Cambridge University Press, 2002).

[7] A. Coco and G. Russo, Finite-difference ghost-point multigrid methods on cartesian grids for elliptic problems in arbitrary domains, J. Comput. Phys. **241**, 464 (2013).

[8] M. Oevermann, C. Scharfenberg, and R. Klein, A sharp interface finite volume method for elliptic equations on cartesian grids, J. Comput. Phys. **228**, 5184 (2009).

[9] J. Papac, F. Gibou, and C. Ratsch, Efficient symmetric discretization for the poisson, heat and stefan-type problems with robin boundary conditions, J. Comput. Phys. **229**, 875 (2010).
